# Yeast-Free Doughs by *Zymomonas mobilis*: Evaluation of Technological and Fermentation Performances by Using a Metabolomic Approach

**DOI:** 10.3390/microorganisms8060792

**Published:** 2020-05-26

**Authors:** Lorenzo Nissen, Manuela Rollini, Claudia Picozzi, Alida Musatti, Roberto Foschino, Andrea Gianotti

**Affiliations:** 1 CIRI-Interdepartmental Centre of Agri-Food Industrial Research, Alma Mater Studiorum—University of Bologna, P.za G. Goidanich 60, 47521 Cesena, Italy; lorenzo.nissen@unibo.it (L.N.); andrea.gianotti@unibo.it (A.G.); 2 DeFENS-Department of Food, Environmental and Nutritional Sciences, Università degli Studi di Milano, Via Mangiagalli 25, 20133 Milano, Italy; manuela.rollini@unimi.it (M.R.); claudia.picozzi@unimi.it (C.P.); roberto.foschino@unimi.it (R.F.); 3 DiSTAL-Department of Agricultural and Food Sciences, Alma Mater Studiorum—University of Bologna, V.le Fanin 50, 40127 Bologna, Italy

**Keywords:** *Zymomonas mobilis*, leavening performance, yeast-free dough, SPME-GC-MS, multivariate analysis

## Abstract

This research focuses on the leavening performances and development of volatile compounds of three strains of *Zymomonas mobilis* in the production of yeast-free doughs. *Z. mobilis* DSM 3580, 424, and 473 were used in doughs supplemented with glucose and with or without NaCl. *Z. mobilis* produced about 10 mg ethanol/g dough, with maximum dough volumes (640–680 mL) being reached after 2 h leavening. NaCl addition postponed this parameter up to 6 h. Among organic acids, hexanoic acid resulted the highest produced compound; DSM 424 and 473 formed more propanoic, butanoic and pentanoic acid, being both negatively affected by NaCl. Esters were mainly discriminated on NaCl addition, with octanoic acid (DSM 3580), butanoic acid (DSM 424), and propanoic acid (DSM 473) ethyl esters as main components. DSM 3580 specifically produced 2-heptanal, DSM 424 2-hexadecenal, (E) and DSM 473 octanal, while DSM 424 and DSM 473 produced 2-butanone-4-hydroxy better than DSM 3580. *Z. mobilis* unique signatures were the production of nonanoic and undecanoic acids, 2-hexadecenal, (E), L(+)-tartaric acid diethyl ester and 3-decen-5-one, 4-methyl, (E). This outcome can pave the way for using *Z. mobilis* in baking goods, providing innovation possibilities in the area of yeast-free leavened products.

## 1. Introduction

Adverse reactions from the ingestion of foods containing baker’s yeast are increasing in Western people. Although still debatable, ASCAs (Anti-*Saccharomyces cerevisiae* antibodies) recognize the parietal components of *S. cerevisiae* cells and are often found in inflammatory states of the human intestinal tract for pathologies such as irritable bowel syndrome (IBS) and Crohn’s disease (CD) [1,2]. Since dietary therapy excludes yeast-containing foods for most patients, interest in the replacement of *S. cerevisiae* in bread-making and other fermentation processes is growing. For fulfilling this need, scientific research has been focused on the leavening performance of *Zymomonas mobilis* [3,4]. Due to its similarity with the fermentative metabolism of *S. cerevisiae*, this Gram-negative bacterium can be considered an attractive alternative to yeast.

The main drawback is that this bacterium can use only glucose, fructose and sucrose, carbohydrates present in flours at very limited concentrations, while maltose remains unconsumed. Based on these considerations, Musatti et al. [5,6] set up a yeast-free bakery product leavened with its unconventional association with *Lactobacillus sanfranciscensis* exploiting the maltose hydrolytic activity of the LAB. Nevertheless, results showed that a controlled addition of glucose to the dough generated better results than those obtained with the co-culture of *L. sanfranciscensis*, which strongly acidified the dough slowing down the growth of *Z. mobilis*. Recent results also evidenced that sucrose can be successfully used by *Z. mobilis* to leaven a dough, with improved gas production and retention capacity [7].

The ability of *Z. mobilis* to develop volatile compounds in a dough has not been studied yet. Metabolomic profiling has been proposed as a promising tool to assess the traceability and quality of fermented food such as bread [8,9,10], dairy products [11], beverages [12,13], and other traditional fermented foods [14].

In this study, a metabolomic approach to investigate technological, microbiological and volatile descriptors is suggested. Three strains of *Z. mobilis* were comparatively used to ferment wheat dough formulations with different content of glucose and NaCl.

## 2. Materials and Methods

### 2.1. Z. mobilis Strains Maintenance and Biomass Production

Three *Zymomonas mobilis* strains were used, i.e., DSM 424, 473 and 3580 (DSMZ: Deutsche Sammlung von Mikroorganismen und Zellkulturen GmbH, Braunschweig, Germany), maintained and stored as reported by Musatti et al. [5].

Each strain was cultured in 1 L flasks employing IC G20 liquid medium [6] added with 5 g/L of meat extract (VWR International PBI srl, Milan, Italy). Biomass was collected by centrifugation and growth determined as previously reported [4].

### 2.2. Flour Characterization

Dough samples were produced using wheat flour type “00 Rapid” (Molino Colombo s.a.s., Paderno d’Adda, Italy; technical data sheet in Appendix A, Table A1). Mixing properties were assessed in samples of flour only (F), in presence of glucose (VWR International) (3 g/100 g flour, FG), or of glucose and NaCl (Sigma Aldrich, St. Louis, MO, USA) (3 and 1 g/100 g flour, respectively, FGN), by means of a Brabender^®^ Farinograph (Brabender OHG, Germany; 300 g chamber, 30 °C, ISO 5530-1:2013). Arrival time (min) and dough stability (min) were assessed through the Brabender^®^ Farinograph 2.5.2 software.

### 2.3. Dough Production, Evaluation of Volume Increase, and Total CO_2_ Production

According to the presence of glucose, the presence/absence of NaCl and the three *Z. mobilis* strains, eight dough models were produced and tested in duplicate (Table 1). Doughs were prepared using a food mixer (CNUM5ST, Bosch, Stuttgart, Germany, 6 min at speed 1): *Z. mobilis* (1.5 mg/g dough, approximately 5 × 10^8^ CFU/g dough) as well as glucose and NaCl when required by the formulation, were suspended in distilled water and then added to the flour.

Each dough was portioned: 250 g were inserted into a 1 L graduate cylinder to evaluate dough volume increase (mL, starting from 210 ± 20 mL) and 25 g into a double chamber flask connected with a graduate burette to calculate CO_2_ production (mL) [4]. Portions were all incubated at 26 °C (± 1 °C) for up to 6 h leavening and monitored every 30 min. Time course of the parameters was fitted employing the DMFit 3.5 software (Combase, University of Tasmania and the USDA Agricultural Research Service) obtaining the following indices: gas production rate (mL/min), lag leavening time (min) and total CO_2_ production (mL).

### 2.4. Dough Chemical Characterization, pH Monitoring and Microbial Counts

At 0, 2, 4, 6 h of leavening, dough pH was measured on the dough samples by specific probe (pH-meter Eutech Instruments pH 510, Toronto, Canada). At the same intervals, 5–8 g of dough were decimally suspended and homogenized. Maltose, glucose and ethanol concentrations were determined through an HPLC system (L 7000, Merck Hitachi) equipped with refractive index detector [6]. *Z. mobilis*, Aerobic Mesophilic Count (AMC) and yeasts and moulds (Y&M) counts were carried out as previously stated [7] and reported in log colony forming units/g of dough (log CFU/g) as means and standard deviations values.

### 2.5. Volatile Compounds Determination by Solid-Phase Microextraction-Gas Chromatography-Mass Spectrometry (SPME-GC-MS)

Evaluation of volatile organic compounds (VOCs) was carried out on an Agilent 7890A Gas Chromatograph (Agilent Technologies, Santa Clara, CA, USA) coupled to an Agilent Technologies 5975 mass spectrometer operating in the electron impact mode (ionization voltage of 70 eV), equipped with a Chrompack CP-Wax 52 CB capillary column (50 m length, 0.32 mm ID) (Chrompack, Middelburg, The Netherlands). The SPME-GC-MS (solid phase micro-extraction gas chromatography–mass spectrometry) protocol and the identification of volatile compounds were done according to previous reports, with minor modifications [9,10]. Briefly, before each head space sampling, the fiber was exposed to the GC inlet for 10 min for thermal desorption at 250 °C in a blank sample. The samples were then equilibrated for 10 min at 40 °C. The SPME fiber was exposed to each sample for 40 min and the fiber was inserted into the injection port of the GC for a 10 min sample desorption. The temperature program was: 50 °C for 0 min, then programmed at 1.5 °C/min to 65 °C, and finally at 3.5 °C/min to 220 °C, which was maintained for 20 min. Injector, interface, and ion source temperatures were 250, 250, and 230 °C, respectively. Injections were carried out in splitless mode, and helium (3 mL/min) was used as carrier gas. The identification of molecules was carried out by comparing their retention times with those of pure compounds (Sigma, USA) and confirmed by searching mass spectra in the available databases (NIST version 2005 and Wiley version 1996) and literature [9,10]. VOCs were relatively quantified in percentage from two independent experiments and two replicates.

### 2.6. Statistical Analysis

Samples were prepared in duplicate and from two independent experiments. Physiological and process data were managed by Statgraphics Centurion (v. 18, Statistical Graphics Corp., Herndon, VA, USA). One-way analysis of variance (ANOVA) was performed using the Least Significant Difference (LSD) test to compare the sample means, considering differences significant at *P* < 0.01.

Statistical analyses regarding the volatilome and its correlations with physiological parameters were performed using TIBCO Statistica 8.0 (Tibco Inc., Palo Alto, CA, USA). Normality, homoscedasticity, and variance were achieved in accordance to Granato et al. [15]. Principal component analysis (PCA), K-mean clustering, Spearman rank correlations, and two-way joining heatmap were used to study the relationship between variables [12,13]. Targeted categorical ANOVA (*P* < 0.01) was employed to check contributions promoted by each strain and dough formula. For post-hoc testing, a Tukey’s HSD (honestly significant difference) was employed. For PCA and Spearman rank correlations, data were normalized using the mean centering method.

## 3. Results and Discussion

### 3.1. Microbial and Chemical Characterizations of Non-Inoculated Dough

Negative control doughs were prepared without *Z. mobilis* strains. When glucose or the combination glucose-NaCl were added to the flour, a lower water absorption was attained (53.3 and 51.4% respectively vs. 55.9%) (Table 1). The arrival time was slightly affected by the addition of glucose while dough stability was strongly delayed when NaCl was present (16.6 vs. 9.5 min). Similar results were evidenced by McCann & Day [16], indicating that the hydration of gluten proteins and the development of gluten matrix in doughs were considerably delayed in the presence of NaCl.

AMC, Y&M counts, and pH showed no variation during leavening (Table 2, negative control). HPLC analyses showed a progressive increase in the maltose content from about 10 to 18 mg/g due to activity of endogenous flour amylases [6]. Glucose remained unconsumed either in FG or FGN doughs. No increase in dough volume was observed in all the non-inoculated samples and CO_2_ production was always lower than 2 mL after 6 h leavening (data not shown).

### 3.2. Microbial, Chemical and Technological Characterizations of Z. mobilis Inoculated Doughs

AMC and Y&M counts in inoculated doughs remained always around their initial values: no differences were noticed among strains or due to the presence of NaCl (Table 2). AMC reached values in the range 2.80–4.12 log CFU/g, while Y&M counts between 2.20 and 3.40 log CFU/g. Similarly, *Z. mobilis* counts remained unchanged during leavening, with values ranging from 8.2 to 9.2 log CFU/g, highlighting an efficient fermentative performance.

All tested strains generated a slight but significant pH decrease respect to the controls, from 2 h leavening onward. DSM 3580 produced the strongest pH dropping, reaching values of 4.82 ± 0.14 at the end of leavening in presence of NaCl. The analyses of organic acids production have been extensively investigated in Section 3.3.1.

Glucose, initially present at a concentration of about 20 mg/g dough, was almost consumed in the first 2 h (residual 0.90 mg/g dough). When NaCl was added, DSM 424 left 50% of the residual glucose unconsumed (10.23 mg/g); DSM 473 and 3580 delayed glucose consumption mainly between 4 and 6 h, with a residual glucose concentration of 5.73 and 2.77 mg/g, respectively. As expected, maltose slightly increased in all samples.

Ethanol reached values of around 10 mg/g dough when only glucose was added; in presence of NaCl, fermentative performances significantly reduced to 6–7 mg ethanol/g dough for DSM 424 and 3580, while remained quite similar for DSM 473 (9.1 mg ethanol/g).

**Figure 1:**
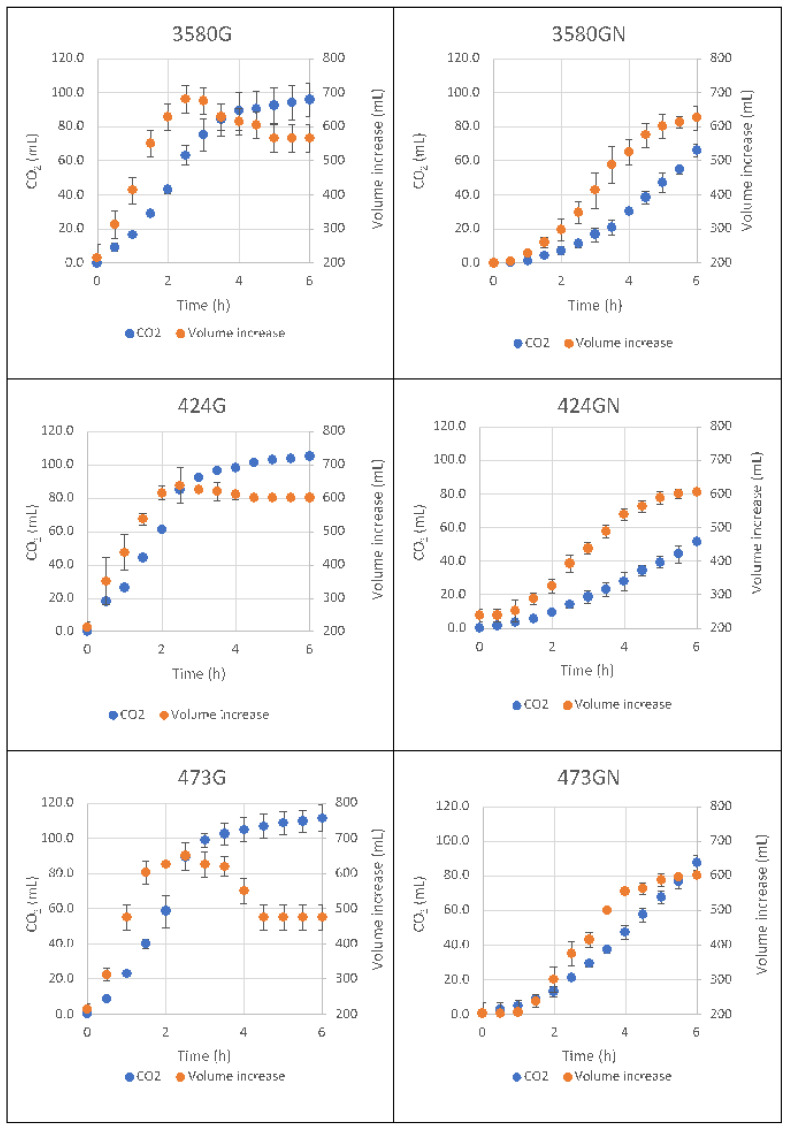
Time course of dough volume increase (mL) and CO_2_ production (mL) in leavening trials carried out with *Z. mobilis* DSM 424, 3580 and 473, comparatively; doughs added with glucose (first column, G) or with glucose and NaCl (second column, N).

As regards the technological performances (Figure 1), DSM 424 and 3580 reached the maximum dough volume at 2.5 h leavening (640-680 mL), while with NaCl the dough attained comparable values only after 6 h. Ethanol was lower in NaCl samples with residual glucose of about 5 mg/g, which instead was totally consumed in the unsalted dough. DSM 473 showed lower rising performance when NaCl was added. Analysis of the leavening kinetics (Table 3) evidenced that, in the presence of glucose, DSM 473 possessed the fastest leavening rate (46.15 ± 3.70 mL/h). When NaCl was also added, this strain still maintained the shortest lag time (1.70 ± 0.17 h), even if it evidenced the highest kinetic reduction together with DSM 3580 (−43% and −48%, respectively).

Musatti et al. [7] reported that with a lower inoculum (7 vs. 8 log CFU/g dough used here) and in presence of sucrose, *Z. mobilis* DSM 424 showed a significantly higher lag time of 7.12 h; in the same condition, the benchmark yeast *S. cerevisiae*, evidenced a leavening rate of around 15 mm/h and a lag time of around 3 h.

Homayouni & Kasaie [18] tested the fermentation performance of four commercial *S. cerevisiae* strains inoculated at 8 log CFU/g dough with NaCl 0.9 g/100 g flour, reporting CO_2_ production variable from 139 to 163 mL/5 g dough.

### 3.3. Volatilome Anlysis

**Figure A1:**
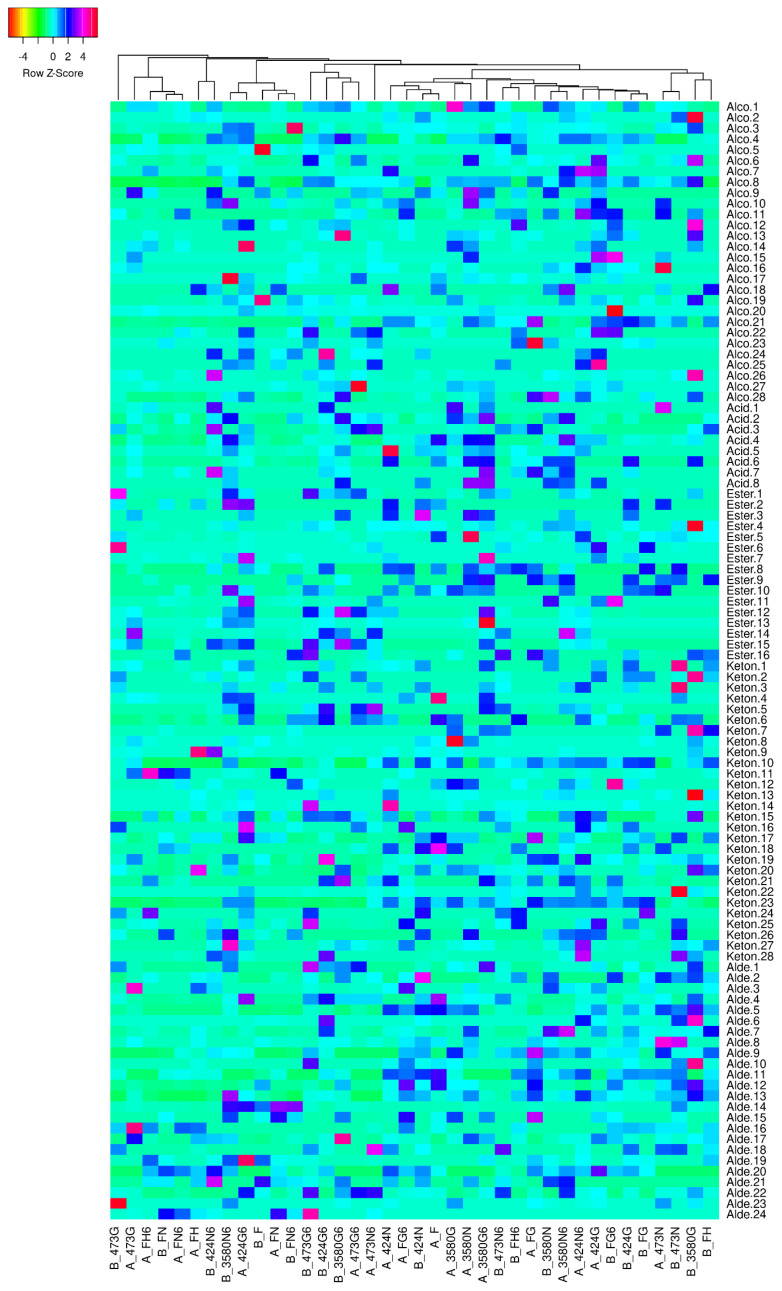
Heatmap of relative quantification of significant molecules (*P* < 0.05). Sample abbreviations: A_3580G and B_3580G = *Z. mobilis* 3580 not leavened doughs added with glucose; A_3580N and B_3580N = A_3580G and B_3580G added with NaCl; A_424G and B_424G = *Z. mobilis* 424 not leavened doughs added with glucose; A_424N and B_424N = A_424G and B_424G added with NaCl; A_473G and B_473G = *Z. mobilis* G473 not leavened doughs added with glucose; A_473N and B_473N = A_473G and B_473G added with NaCl; A_F and B_F = Flours; A_FG and B_FG = Not inoculated control dough added with glucose; A_FH and B_FH = Not inoculated control dough; A_FN and B_FN = A_FG and B_FG added with NaCl; A_3580G6 and B_3580G6 = A_3580G and B_3580G leavened for 6 h; A_3580N6 and B_3580N6 = A_3580N and B_3580N leavened 6 h; A_424G6 and B_424G6 = A_424G and B_424G leavened 6 h; A_424N6 and B_424N6 = A_424N and B_424N leavened 6 h; A_473G6 and B_473G6 = 473G and B_473G leavened 6 h; A_473N6 and B_473N6 = A_473N and B_473N leavened 6 h; A_FG6 and B_FG6 = A_FG and B_FG leavened 6 h; A_FH6 and B_FH6 = A_FH and B_FH leavened 6 h; A_FN6 and B_FN6 = A_FN and B_FN leavened 6 h. VOCs abbreviations: Alco 1 = 1-butanol, 2-methyl; Alco 2 = 3-butynol; Alco 3 = 1-pentanol; Alco 4 = 1-hexanol; Alco 5 = 3-cyclohexenol, 4-methyl; Alco 6 = 2-heptanol; Alco 7 = 1-heptanol, 4-methyl; Alco 8 = 1-octanol; Alco 9 = 1-octanol-2-butyl; Alco 10 = 1-octanol, 3,7-dimethyl; Alco 11 = 2,7-octadienol; Alco 12 = 6-nonenol; Alco 13 = 1-decanol; Alco 14 = 1-decanol, 2-methyl; Alco 15 = 1-undecanol; Alco 16 = 2-decenol; Alco 17 = 1-dodecanol; Alco 18 = Isotridecanol; Alco 19 = Tetradecanol; Alco 20 = 1,4-butenediol; Alco 21 = 1-heneicosanol; Alco 22 = 2,4-decadienol; Alco 23= Isophytol; Alco 24 = 1-pentadecanol; Alco 25 = 1-hexadecanol; Alco 26 = 2-phenylethanol; Alco 27= 3-furanmethanol; Alco 28 = Benzyl alcohol; Acid 1= Propanoic acid; Acid 2 = Acetic acid; Acid 3= Pentanoic acid; Acid 4 = Hexanoic acid; Acid 5 = Heptanoic acid; Acid 6 = Octanoic acid; Acid 7 = Nonanonic acid; Acid 8 = Undecanoic acid; Ester 1= Propanoic acid ethyl ester; Ester 2 = 2-chloropropionic acid octadecyl ester; Ester 3 = Bromoacetic acid undec-2-enyl ester; Ester 4= Acetic acid trichloro nonyl ester; Ester 5 = Butanoic acid ethyl ester; Ester 6 = Valeric acid but-2-enyl ester; Ester 7 = Hexanoic acid ethyl ester; Ester 8= Heptanoic acid dec-2-enyl ester; Ester 9 = Octanoic acid ethyl ester; Ester 10 = L(+)-tartaric acid diethyl ester; Ester 11 = Pentadecanoic acid ethyl ester; Ester 12 = Hexadecanoic acid ethyl ester; Ester 13 = Octadec-2-enoic acid ethyl ester; Ester 14 = Ethyl Oleate; Ester 15 = Linoleic acid ethyl ester; Ester 16 = Thiopheneacetic undec-2-enyl ester; Keton 1 = Ethanone; Keton 2 = Acetophenone; Keton 3= Propanone; Keton 4 = 3-butanone, 2-methyl; Keton 5 = 2-butanone-3-hydroxy; Keton 6 = 3-buten-2-one, 3-methyl; Keton 7= 3-ethylcyclopentanonone; Keton 8= Hex-3-en-2-one; Keton 9 = Cyclohexanone, 2-butyl; Keton 10 = 4-(Tert-butyl)-cyclohexanone; Keton 11 = 4-isopropyl-2-cyclohexenone; Keton 12 = 2-heptanone; Keton 13 = 3-heptanone, 2,4-dimethyl; Keton 14 = 5-hepten-2-one, 6-methyl; Keton 15 = 2-octanone; Keton 16 = 3-octanone, 2,2-dimethyl; Keton 17 = dimethyl octenone; Keton 18 = gamma nonalactone; Keton 19 = octadien-3-one; Keton 20 = 3,5-octadienone, (E,E); Keton 21 = Nonanone; Keton 22 = Decanone; Keton 23 = 3-decen-5-one, 4-methyl; Keton 24 = Tridecadione; Keton 25 = Pentadecanone; Keton 26 = Benzalacetone; Keton 27 = Furanone; Keton 28 = Perhydroazulenone; Alde 1 = Butanal; Alde 2 = butanal, 3-methyl; Alde 3= 2-butenal, 3-methyl; Alde 4 = Pentanal; Alde 5 = Hexanal; Alde 6 = 2-hexenal, (E); Alde 7 = Heptanal; Alde 8 = 2-heptenal, (E); Alde 9 = 2,4-heptadienal; Alde 10 = Octanal; Alde 11 = 2-octenal, (Z); Alde 12 = Nonanal; Alde 13 = 2-nonenal; Alde 14 = 2,4-nonadienal; Alde 15 = Decanal; Alde 16 = 2-dodecenal, (E); Alde 17 = 2,4-decadienal; Alde 18 = 2,4-dodecadienal, (E,E); Alde 19 = 2 hexadecenal, (E); Alde 20 = 9-octadecenal, (E,E); Alde 21 = Furaldehyde; Alde 22 = Benzaldehyde; Alde 23 = Furancarboxaldehyde; Alde 24 = Hydroxybenzaldehyde.

Through SPME GC-MS, among 36 duplicated cases (n = 72), 236 molecules were identified: on average, 75 were relatively quantified in unleavened doughs (t0), while 120 in leavened samples (6 h). For a landscape description of the volatilome, a data set of 104 significant molecules (ANOVA at *P* < 0.05) was generated and expressed as a quantification heatmap (Figure A1, Appendix A). Multivariate analyses (PCA and K-means clustering) was achieved from super-normalized data sets organized by different chemical classes of VOCs, i.e., organic acids, organic acid esters, aldehydes, and ketones, but not from alcohols. To study correlations, Spearman rank analysis (*P* < 0.05) results were obtained from single data sets of VOCs, and normalized values of nine dependent variables obtained from microbial growth, pH reduction, dough volume increase, sugars consumption, CO_2_, and ethanol production. Lastly, to address the specific contributes on VOCs production by independent variables, targeted ANOVA (*P* < 0.01) was performed, including two categorical predictors, such as *Z. mobilis* inoculated strains (plus non-inoculated dough as negative control) and dough formulations (F, FG, FGN).

#### 3.3.1. Multivariate Analysis of Organic Acids

**Figure 2:**
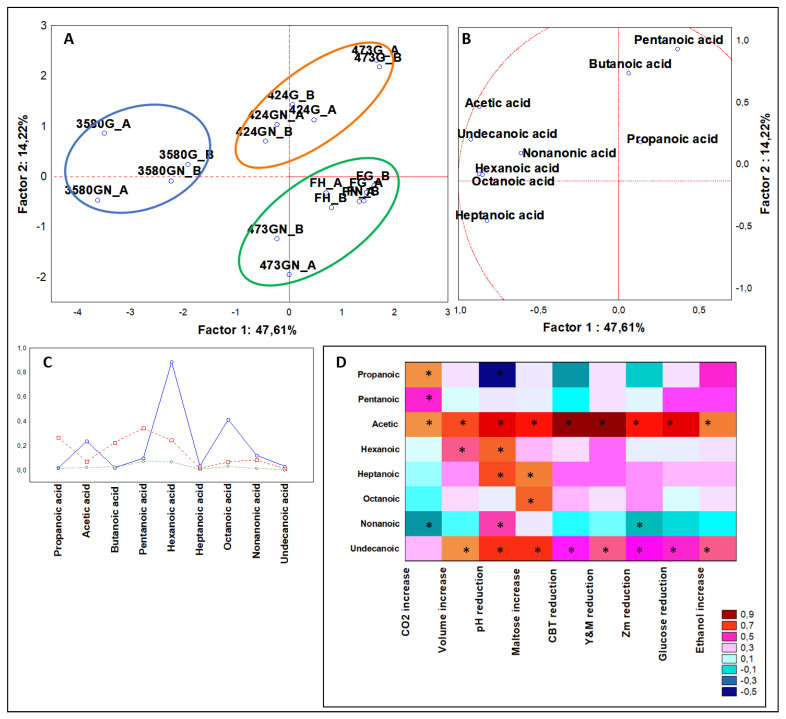
(**A**) PCA of cases and (**B**) variables on organic acids; (**C**) K-means clustering; (**D**) Spearman rank correlations on alcohols. * *P* < 0.05. Sample abbreviations: FH_A and FH_B = Not-inoculated leavened doughs; FG_A and FG_B = FH_A and FH_B added with glucose; FGN_A and FGN_B = FG_A and FG_B added with NaCl; 3580G_A and 3580G_B = *Z. mobilis* 3580 leavened doughs added with glucose; 3580GN_A and 3580GN_B = 3580G_A and 3580G_B added with NaCl; 424G_A and 424G_B = *Z. mobilis* 424 leavened doughs added with glucose; 424GN_A and 424GN_B = 424GN_A and 424GN_B added with NaCl; 473G_A and 473G_B = *Z. mobilis* G473 leavened doughs added with glucose; 473GN_A and 473GN_B = 473G_A and 473G_B added with NaCl. CBT reduction = reduction in AMC (Aerobic Mesophilic Counts); Y&M reduction = reduction in Yeast and Molds; Zm reduction = reduction in *Z. mobilis.*

Organic acids in bread making are principally produced during fermentation and give a large contribute both sensorially and nutritionally. An equilibrium in the content of organic acids is important because some, like short chain organic acids, are considered prebiotics and host health-related compounds, but confers a stinky rancid and putrid taste [10,13]. From ANOVA that included leavened samples (n = 36), nine molecules belonging to the class of organic acids (C2–C11) resulted statistically significant (*P* < 0.05). PCA spread cases and variables on a plane and based on the distances to the plot center defined the observed differences (Figure 2A,B). The plane generated by factor 1 of 47.61% and factor 2 of 14.22% resulted robust. Applying K-means clustering on PCA loadings, three clusters were drawn (Figure 2C).

Cluster 1 included doughs all leavened by *Z. mobilis*, described by top concentration of hexanoic (remarkably the highest), octanoic, acetic and nonanoic acids. Among *Z. mobilis* strains, DSM 3580 produced the double amount of acetic acid and four-times the amount of hexanoic and octanoic acids.

Cluster 2 contained six cases (n = 12) including four cases leavened by DSM 424 and two by DSM 473. This cluster was described by the highest quantity of pentanoic, propanoic, and butanoic acids, reported in decreasing order. Moreover, samples leavened with DSM 424 were grouped together and the addition of NaCl seemed irrelevant. Otherwise, doughs inoculated with DSM 473 were distant up left from other members, described by higher levels of pentanoic acid.

From the Spearman rank analyses (Figure 2D) the correlation results defined that the amount of propanoic acid was positively linked to CO_2_ increase and negatively to pH reduction, while the amount of nonanoic acid was correlated in an opposite fashion.

**Figure A2:**
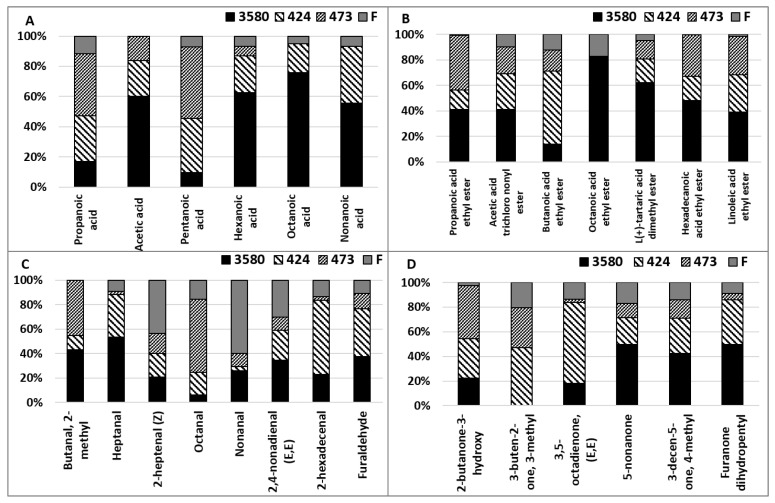
ANOVA plots with categorical predictors set on *Z. mobilis* strains (*P* < 0.01), relative to; (**A**) organic acids; (**B**) esters of organic acids; (**C**) aldehydes; (**D**) ketones. F = not-inoculated leavened doughs; 3580 = *Z. mobilis* 3580 leavened doughs; 424 = *Z. mobilis* 424 leavened doughs; 473 = *Z. mobilis* 473 leavened doughs.

**Figure A3:**
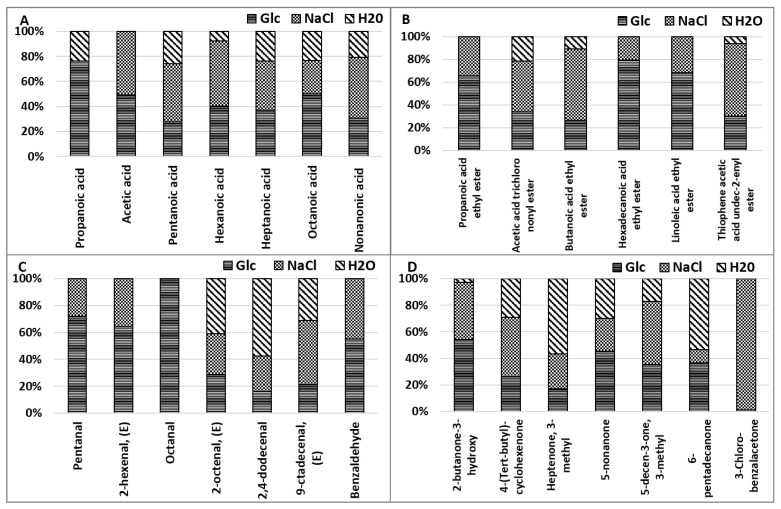
ANOVA plots with categorical predictors set on the presence or absence of NaCl (*P* < 0.01), relative to; (**a**) organic acids; (**b**) esters; (**c**) aldehydes; (**d**) ketones. F = not-inoculated leavened doughs; Glc = leavened doughs added with glucose; NaCl = leavened doughs added with NaCl; H_2_O = leavened doughs not inoculated and without glucose or NaCl.

From the targeted analysis categorized for strains (Figure A2A, Appendix A), six organic acids were significant (*P* < 0.01) with DSM 3580 producing roughly the 60% of each total amount of acetic, hexanoic, octanoic, and nonanoic acids. DSM 473 was the best producer of propanoic and pentanoic acids, generating approximately 40% of each of these molecules. Instead, taking into consideration the dough formulation category (Figure A3A, Appendix A), NaCl addition significantly (*P* < 0.01) increased pentanoic, hexanoic and nonanoic acids were produced more abundantly, while propanoic acid was not produced at all. The presence of short chain fatty acids, such as acetic, propanoic, and butanoic, and of medium chain fatty acids, e.g., pentanoic, hexanoic, octanoic, and nonanoic, is similarly found in wheat doughs fermented by *S. cerevisiae* and in sourdough, contributing largely to the aromatic profile of baker’s yeast fermented doughs from different flours [19,20,21]. Besides, these compounds give an important beneficial contribute to host well-being, mainly talking about the prebiotic effect [22] and the role in cell homeostasis [23,24]. The most of short and medium chain organic acids have a typical rancid-like aroma, but few are pleasant. Considering the unique organic acids of *Z. mobilis* leavened doughs contributing to the aroma profile, pleasant issues are described for nonanoic acid as waxy, dirty, and cheesy [25], and undecanonic acid as creamy, cheese like with a coconut nuance [26].

#### 3.3.2. Multivariate Analysis of Organic Acid Esters

During dough fermentation many organic acid esters are produced. Considering their sensorial attributes some are desirable, because generally confer mild sweet fruity traits, like ethyl propionic or ethyl butyric acid esters, but some could present an excessive sweetness and results unpleasant such as 2-mehtyl-butyl acetic acid ester that has a scents described as that of overripe fruit [20].

**Figure 3:**
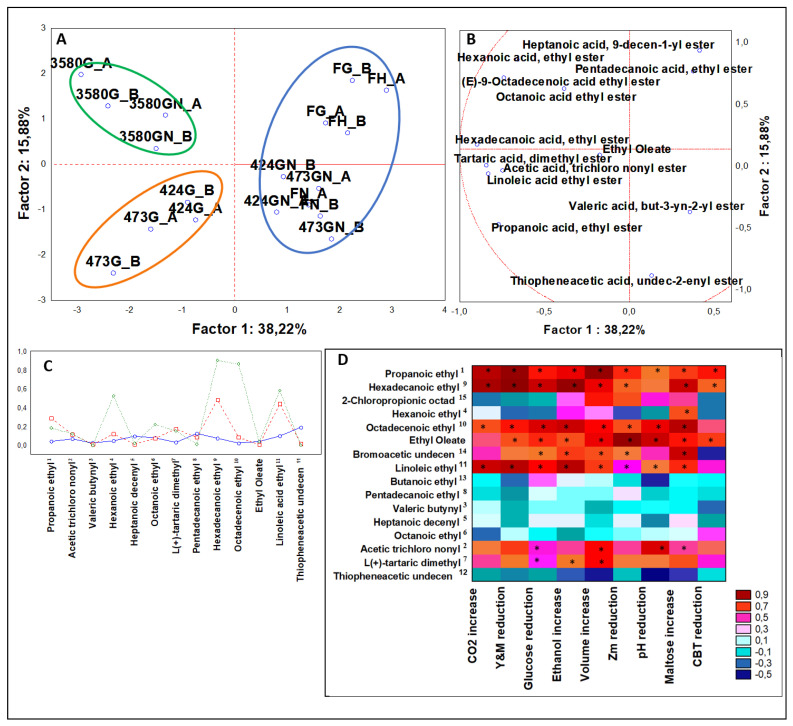
(**A**) PCA of cases and (**B**) variables on esters; (**C**) K-means clustering; (**D**) Spearman rank correlations on aldehydes. * *P* < 0.05. Sample abbreviations: FH_A and FH_B = Not-inoculated leavened doughs; FG_A and FG_B = FH_A and FH_B added with glucose; FGN A and FGN B = FG A and FG B added with NaCl; 3580G_A and 3580G_B = *Z. mobilis* 3580 leavened doughs added with glucose; 3580GN_A and 3580GN_B = 3580G_A and 3580G_B added with NaCl; 424G_A and 424G_B = *Z. mobilis* 424 leavened doughs added with glucose; 424GN_A and 424GN_B = 424GN_A and 424GN_B added with NaCl; 473G_A and 473G_B = *Z. mobilis* G473 leavened doughs added with glucose; 473GN_A and 473GN_B = 473G_A and 473G_B added with NaCl. ^1^ Propanoic acid ethyl ester; ^2^ Acetic acid trichloro nonyl ester; ^3^ Valeric acid but-2-enyl ester; ^4^ Hexanoic acid ethyl ester; ^5^ Heptanoic acid dec-2-enyl ester; ^6^ Octanoic acid ethyl ester; ^7^ L(+)-tartaric acid diethyl ester; ^8^ Pentadecanoic acid ethyl ester; ^9^ Hexadecanoic acid ethyl ester; ^10^ Octadec-2-enoic ethyl ester; ^11^ Linoleic acid ethyl ester; ^12^ Thiopheneacetic undec-2-enyl ester; ^13^ Butanoic acid ethyl ester; ^14^ Bromoacetic undec-2-enyl ester; ^15^ 2-chloropropionic acid octadecyl ester. CBT reduction = reduction in AMC (Aerobic Mesophilic Counts); Y&M reduction = reduction in Yeast and Molds; Zm reduction = reduction in *Z. mobilis.*

From ANOVA that included leavened samples (n = 36), 13 organic acid esters (C2-C18) were statistically significant (*P* < 0.05). With respect to the PCA (Figure 3A,B), three clusters were obtained (Figure 3C). Unsalted doughs leavened by DSM 424 and 473 were included in cluster 2, described by the highest abundance of propanoic acid ethyl and L(+)-tartaric acid diethyl esters.

Samples inoculated with DSM 3580 were confined in cluster 3, independently from the dough formulation. DSM 3580 leavened doughs were characterized by top abundance of hexanoic, octanoic, and hexadecanoic acid ethyl esters.

Considering Spearman Rank analysis (Figure 3D), a set of compounds was positively correlated to the physiological variables, i.e., propanoic, hexadecanoic, linoleic, and (E)-9-octadecenoic acid ethyl esters. Oppositely, a set of esters was inversely correlated to almost all variables, butanoic, octanoic, and pentadecanoic acid ethyl esters. In fact, in bakery products, as some esters are generated by the increasing amount of alcohols and organics acids during fermentation, some others are originated by an early lipid oxidation of the matrix depending from the initial fatty acid [27].

The targeted ANOVA categorized for strains (Figure A2B, Appendix A) showed that DSM 3580 produced the 80% of total octanoic acid ethyl and 60% of total L(+)-tartaric acid diethyl esters. DSM 424 formed approximately 60% of total butanoic acid ethyl ester, while DSM 473 produced 45% of propanoic acid ethyl ester. With respect to the category of formulations (Figure A3B, Appendix A), the addition of NaCl generated almost 70% of butanoic acid ethyl ester and more than 65% of thiophene acetic acid undec-2-enyl ester, but just the 20% of hexadecanoic acid ethyl ester.

Production of ester compounds by *Z. mobilis* strains resembles the fermentation features carried out by lactic acid bacteria, more than that obtained with *S. cerevisiae* [28,29,30]. Considering the unique organic acid esters of *Z. mobilis* leavened doughs contributing to the aroma profile, L(+)-tartaric acid diethyl ester is described as earth and fruity tasting [31].

#### 3.3.3. Multivariate Analysis of Aldehydes

**Figure 4:**
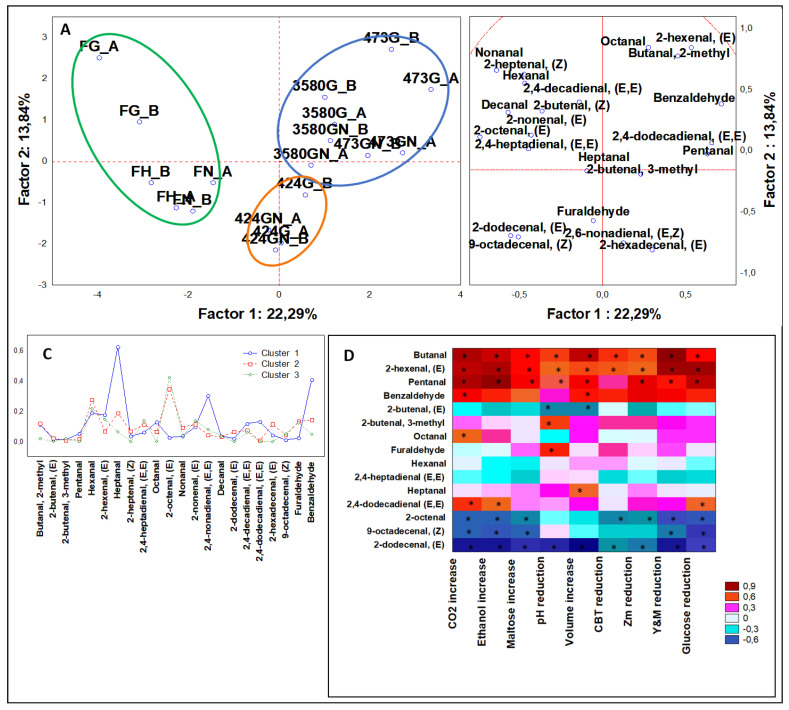
(**A**) PCA of cases and (**B**) variables on aldehydes; (**C**) K-means clustering; (**D**) Spearman rank correlations on ketones. * *P* < 0.05. Sample abbreviations: FH_A and FH_B = Not-inoculated leavened doughs; FG_A and FG_B = FH_A and FH_B added with glucose; FGN A and FGN_B = FG_A and FG_B added with NaCl; 3580G_A and 3580G_B = *Z. mobilis* 3580 leavened doughs added with glucose; 3580GN_A and 3580GN_B = 3580G_A and 3580G_B added with NaCl; 424G_A and 424G_B = *Z. mobilis* 424 leavened doughs added with glucose; 424GN_A and 424GN_B = 424GN_A and 424GN_B added with NaCl; 473G_A and 473G_B = *Z. mobilis* G473 leavened doughs added with glucose; 473GN_A and 473GN_B = 473G_A and 473G_B added with NaCl. CBT reduction = reduction in AMC (Aerobic Mesophilic Counts); Y&M reduction = reduction in Yeast and Molds; Zm reduction = reduction in *Z. mobilis*.

Aldehydes production in leavened dough is a result of microbial fermentation and lipid oxidation [20]. Many aldehydes are desirable because they contribute positively to odor and taste with fruity, floral, and fresh fragrances, like 2-butenal, heptanal or octanal, while others are detrimental expressing a pungent aroma and being toxic at low threshold, like acetaldehyde or benzaldehyde [20]. ANOVA on data derived from inoculated samples (n = 36) gave 22 aldehydes (C2-C18) with statistically significant results (*P* < 0.05). PCA outputs (Figure 4A,B) were grouped in three clusters (Figure 4C).

Cluster 1 contains all samples leavened by DSM 3580 and 473, top-rated in the aldehyde dataset for heptanal, with a load four-fold larger than the runner-up; three other compounds, i.e., benzaldehyde, 2,6-nonadienal (E,E) (four-time more abundant than the cluster with unleavened samples) and 2,4-dodecadienal (E,E) were also evidenced. The former molecule was found more than double compared to the cluster containing samples leavened with the third *Z. mobilis* strain DSM 424. Cluster 2 contained doughs leavened by DSM 424 and nonanal and 2-hexadecenal, (E) represented their unique signature. Cluster 3 was composed by all control samples and described by high levels of 2-hexenal (E), 2-octenal (E), and 2,4-heptadienal (E,E). The presence of these latter compounds is typical of unleavened dough and lipid oxidation of flours [32].

From Spearman rank analysis butanal, 2-hexenal (E), and pentanal showed positive correlations to physiological variables, while 2-octenal, 9-octadecenal, (E), and 2,4-dodecenal inverse correlations. So far, high amount of butanal, 2-hexenal (E), and pentanal were promoted by *Z. mobilis* fermentation and experimental conditions.

Statistical analysis categorized for leavening strains (Figure A2C, Appendix A) showed that DSM 3580 accounted for the 55% of heptanal production in doughs, DSM 424 more than 60% of total 2-hexadecenal (E), and DSM 473 62% of octanal. Instead, investigating formulation category (Figure A3C, Appendix A), it was observed that the addition of NaCl did not produce any octanal, but formed 68% of total 9-octadecenal, (E).

Noteworthy, heptanal and octanal in literature are reported to be generated by *S. cerevisiae* fermentation through oxidation of polyunsaturated fatty acids into free radical peroxides and hydroperoxides, which are then converted in volatile aldehydes [19,33]. Moreover, these compounds in bakery products are contributing largely to the aromatic profile of doughs, heptanal with fresh, green, fatty nuances, and octanal with fruity, floral, and waxy ones [20].

#### 3.3.4. Multivariate Analysis of Ketones

During dough fermentation many ketones are produced, considering their sensorial attributes some are desirable, like 2-butanone-3-hydroxy holding sweet and creamy nuances, others like (Z) -1,5-Octadien-3-one or octen-3-one are unwanted, because even at a very low threshold confers earthy and musty nuances [20].

**Figure 5:**
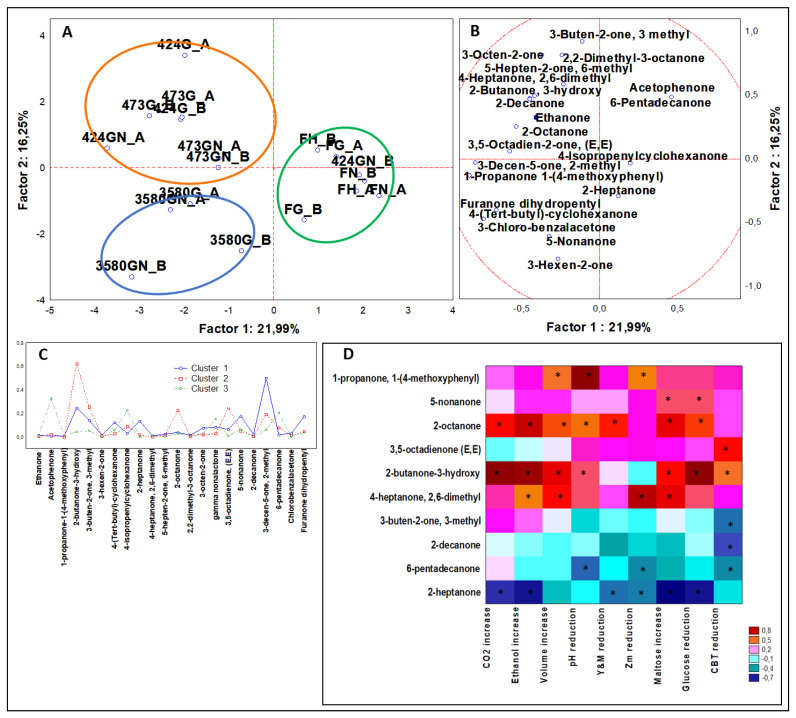
(**A**) PCA of cases and (**B**) variables on ketones; (**C**) K-means clustering; (**D**) Spearman rank correlations on alkanes. * *P* < 0.05. Sample abbreviations: FH_A and FH_B = Not inoculated leavened doughs; FG_A and FG_B = FH_A and FH_B added with glucose; FGN_A and FGN_B = FG_A and FG_B added with NaCl; 3580G_A and 3580G_B = *Z. mobilis* 3580 leavened doughs added with glucose; 3580GN_A and 3580GN_B = 3580G_A and 3580G_B added with NaCl; 424G_A and 424G_B = *Z. mobilis* 424 leavened doughs added with glucose; 424GN_A and 424GN_B = 424G_A and 424G_B added with NaCl; 473G_A and 473G_B = *Z. mobilis* G473 leavened doughs added with glucose; 473GN_A and 473GN_B = 473G_A and 473G_B added with NaCl. CBT reduction = reduction in AMC (Aerobic Mesophilic Counts); Y&M reduction = reduction in Yeast and Molds; Zm reduction = reduction in *Z. mobilis*.

From ANOVA that included inoculated samples (n = 36), 22 ketones (C2–C15) were statistically significant (*P* < 0.05). PCA (Figure 5A,B) outputs were defined by three clusters (Figure 5C).

Doughs leavened by DSM 3580 were grouped in cluster 1, described by the maximum concentration of 3-decen-5-one, 4-methyl, about eight and four-fold more than cluster 2 and cluster 3, respectively. Other descriptors were for example, 4-(Tert-butyl)-cyclohexanone, 2-heptanone, 5-nonanone, and gamma-nonalactone.

Cluster 2 comprised samples fermented by DSM 424 and 473, described by the highest concentrations of 2-butanone-3-hydroxy, 3-buten-2-one, 3-methyl, 3,5-octadiene-2-one (E,E), and 2-octanone. The amount of 2-butanone-3-hydroxy was almost three times higher than that of the cluster containing doughs inoculated by DSM 3580 and about six times more than the cluster with controls. 2-octanone figured a clear signature of the samples of this cluster.

Cluster 3 was set opposite the other two, comprised all controls, and was described by the maximum values of acetophenone, 4-isopropyl-2-cyclohexenone, 3,5-octadien-2-one (E,E), and 6-pentadecanone.

The Spearman correlations significantly found four compounds positively correlated and four others inversely correlated to physiological variables.

Targeted ANOVA categorized for leavening strains (Figure A2D, Appendix A) showed that DSM 3580 produced approximately 52% of total nonanone quantity, DSM 424 addressed more than 65% of total 3,5-octadien-2-one (E,E), while DSM 473 for about 45% of 2-butanone-3-hydroxy. Investigating formulation category (Figure A3D, Appendix A), only with the addition of NaCl was chloro-benzalacetone produced, 3-decen-5-one and 4-methyl amounts improved, while 6-pentadecanone was reduced. Moreover, the abundance of 2-butanone-3-hydroxy was not affected by different dough formulation.

Spearman correlations confirmed that 2-butanone-4-hydroxy was bound mainly to fermentation process driven by *Z. mobilis*, and that was not affected by NaCl. Some compounds are typical addressing the sourdough fermentation, such as gamma nonalactone [29], or the fermentation by *S. cerevisiae*, such as 2-heptanone [34]. In bakery products, 2-hepanone is described to have a fruity, spicy, and soap aroma, while gamma nonalactone is described as having an aroma of coconut and butter [19].

## 4. Conclusions

The present paper relates to the use of *Z. mobilis* as a leavening agent in yeast-free doughs. The three tested strains can be successfully used as alternative leavening agents of baker’s yeast. The addition of NaCl in dough formulation determined a slowing of the rising performance: in this condition, the maximum dough volume was postponed at 6 h leavening.

Compared to non-inoculated doughs, the presence of *Z. mobilis* significantly reduced AMC and Y&M counts. This effect may be mainly attributed to the production of ethanol, although the synthesis of other organic acids with an anti-fungal activity cannot be excluded.

The present study for the first time offers the metabolomic profiles of the aromatic VOCs of doughs formulated in different ways and leavened with different *Z. mobilis* strains. The obtained outcome demonstrated that this bacterial species developed proper fermentation products, such as ethanol, acetic acid and 2-butanone-4-hydroxy, similarly to traditional starter cultures used in bakery applications. *Z. mobilis* strains were also able to address a unique signature in wheat fermented doughs as the production of nonanoic and undecanoic acid, 2-hexadecenal, (E), L(+)-tartaric acid diethyl ester, and 3-decen-5-one, 4-methyl.

In conclusion, this versatile bacterium can confer the positive characteristics of both direct and sourdough fermentation technologies without the hassle of the preparation of such processes, nor the presence of allergenic *S. cerevisiae* antigens.

## Figures and Tables

**Table 1:** Doughs formulation and characterization (F = flour only; FG = flour added with glucose; FGN = flour added with glucose and NaCl).

Dough	Flour (g/100 g_flour_)	Water Absorption (g/100 g_flour_)	Glucose (g/100 g_flour_)	NaCl (g/100 g_flour_)	Arrival Time (min)	Dough Stability (min)
F	100	55.9	-	-	2.3	9.5
FG	100	53.3	3	-	1.6	9.1
FGN	100	51.4	3	1	1.7	16.6

**Table 2:** Mean and standard deviation of microbial and chemical parameters detected in dough samples inoculated or not (negative control) with different *Z. mobilis* strains (DSM 3580, 424 and 473), collected during t0, 2, 4 and 6 h leavening.

Parameter	Time (h)	Dough	DSM 3580	DSM 424	DSM 473	Negative Control (Non-Inoculated)
Aerobic	0	FG	3.75 ± 0.19 ^A^	2.30 ± 0.43 ^B^	4.31 ± 0.38 ^A^	3.49 ± 0.07 ^A^
Mesophilic	2		3.57 ± 0.22	3.18 ± 0.17	4.20 ± 0.18	3.21 ± 0.18
Counts AMC	4		3.48 ± 0.16 ^A^	3.47 ± 0.13 ^A^	4.15 ± 0.47 ^B^	3.42 ± 0.04 ^A^
(log CFU/g)	6		3.18 ± 0.31 ^A^	3.43 ± 0.52 ^A,B^	4.12 ± 0.34 ^B^	3.68 ± 0.03 ^B^
	0	FGN	3.32 ± 0.46 ^A^	2.66 ± 0.51 ^B^	3.96 ± 0.03 ^A^	3.50 ± 0.04 ^A^
	2		2.95 ± 0.43	3.36 ± 0.48	3.94 ± 0.02	3.73 ± 0.09
	4		3.23 ± 0.12 ^A^	3.29 ± 0.13 ^A^	3.81 ± 0.01 ^B^	3.62 ± 0.16 ^A^
	6		2.83 ± 0.07 ^A^	3.12 ± 0.59 ^A,B^	3.56 ± 0.01 ^B^	3.92 ± 0.33 ^B^
Yeasts and	0	FG	3.15 ± 0.64	2.82 ± 0.48	3.20 ± 0.08	3.44 ± 0.11
moulds Y&M	2		2.61 ± 0.29 ^A^	2.80 ± 0.21 ^A^	2.60 ± 0.43 ^A^	3.34 ± 0.06 ^B^
(log CFU/g)	4		2.81 ± 0.38 ^A,B^	2.33 ± 0.89 ^A^	2.76 ± 0.40 ^A,B^	3.34 ± 0.29 ^B^
	6		2.52 ± 0.31 ^A^	2.53 ± 0.18 ^A^	2.66 ± 0.38 ^A^	3.21 ± 0.13 ^B^
	0	FGN	2.76 ± 0.08	2.92 ± 0.63	2.60 ± 0.08	3.39 ± 0.07
	2		2.73 ± 0.11 ^A^	2.60 ± 0.60 ^A^	2.50 ± 0.71 ^A^	3.60 ± 0.28 ^B^
	4		2.83 ± 0.02 ^A,B^	2.66 ± 0.51 ^A^	2.51 ± 0.05 ^A^	3.47 ± 0.14 ^B^
	6		2.27 ± 0.38 ^A^	2.70 ± 0.00 ^A^	2.29 ± 0.16 ^A^	3.47 ± 0.11 ^B^
*Z. mobilis*	0	FG	8.23 ± 0.22	9.18 ± 0.05	8.67 ± 0.47	n.d.*
counts	2		8.20 ± 0.87	8.97 ± 0.55	8.72 ± 0.34	n.d.*
(log CFU/g)	4		8.33 ± 0.15 ^A^	9.33 ± 0.16 ^B^	8.69 ± 0.41 ^A,B^	n.d.*
	6		8.05 ± 0.14 ^A^	8.91 ± 0.57 ^B^	8.62 ± 0.23 ^A,B^	n.d.*
	0	FGN	8.75 ± 0.72	8.23 ± 0.04	9.01 ± 0.30	n.d.*
	2		8.08 ± 0.48	8.33 ± 0.03	8.75 ± 0.05	n.d.*
	4		8.16 ± 0.33 ^A^	8.44 ± 0.06 ^A,B^	8.74 ± 0.36 ^B^	n.d.*
	6		7.61 ± 0.75 ^A^	8.30 ± 0.09 ^A,B^	8.73 ± 0.57 ^B^	n.d.*
pH	0	FG	5.86 ± 0.01	5.88 ± 0.07	5.87 ± 0.03	6.00 ± 0.01
(units)	2		5.61 ± 0.23 ^A^	5.66 ± 0.08 ^A^	5.73 ± 0.04 ^A,B^	5.93 ± 0.01 ^B^
	4		5.44 ± 0.05 ^A^	5.52 ± 0.08 ^A,B^	5.63 ± 0.09 ^B^	5.91 ± 0.01 ^C^
	6		5.39 ± 0.01 ^A^	5.50 ± 0.09 ^B^	5.53 ± 0.00 ^B^	5.92 ± 0.06 ^C^
	0	FGN	5.89 ± 0.00	5.87 ± 0.14	5.87 ± 0.07	6.00 ± 0.00
	2		5.23 ± 0.29 ^A^	5.61 ± 0.01 ^A,B^	5.68 ± 0.08 ^A,B^	5.94 ± 0.03 ^B^
	4		4.92 ± 0.26 ^A^	5.32 ± 0.19 ^A,B^	5.55 ± 0.07 ^B^	5.91 ± 0.01 ^C^
	6		4.82 ± 0.14 ^A^	5.08 ± 0.20 ^B^	5.43 ± 0.04 ^B^	5.90 ± 0.01 ^C^
Glucose	0	FG	20.68 ± 0.53	19.05 ± 2.20	18.24 ± 0.95	22.47 ± 1.88
(mg/g)	2		6.44 ± 1.81 ^A,a^	7.32 ± 1.07 ^A,a^	3.04 ± 1.58 ^A,a^	22.32 ± 2.23 ^B^
	4		1.53 ± 0.81 ^A,a^	0.47 ± 0.29 ^A,a^	0.90 ± 0.51 ^A,a^	22.30 ± 1.91 ^B^
	6		0.30 ± 0.42 ^A,a^	0.72 ± 0.16 ^A,a^	0.56 ± 0.15 ^A,a^	22.40 ± 1.04 ^B^
	0	FGN	22.46 ± 0.02	21.21 ± 1.50	21.44 ± 0.45	21.15 ± 0.19
	2		17.09 ± 0.30 ^A,b^	18.22 ± 0.41 ^A,b^	17.55 ± 0.92 ^A,b^	21.54 ± 0.42 ^B^
	4		14.15 ± 0.46 ^A,b^	14.50 ± 2.27 ^A,b^	10.23 ± 1.16 ^A,b^	21.59 ± 1.44 ^B^
	6		5.73 ± 2.06 ^A,b^	10.23 ± 1.04 ^A,b^	2.77 ± 1.68 ^A,b^	21.65 ± 1.26 ^B^
Maltose	0	FG	10.33 ± 0.24	9.81 ± 1.12	10.78 ± 0.64	10.50 ± 1.43
(mg/g)	2		15.25 ± 1.24 ^a^	14.54 ± 0.91^a^	15.92 ± 0.36 ^a^	13.53 ± 0.83
	4		17.97 ± 2.81	17.59 ± 0.83	18.59 ± 1.39	15.61 ± 1.01
	6		20.74 ± 0.93	20.19 ± 1.93	20.37 ± 0.43	18.01 ± 0.70
	0	FGN	9.82 ± 0.20	9.66 ± 0.18	7.95 ± 0.81	9.38 ± 0.15
	2		12.99 ± 0.26 ^b^	13.24 ± 0.27^b^	12.90 ± 0.42 ^b^	13.48 ± 0.30
	4		17.88 ± 1.61	15.49 ± 0.71	15.77 ± 0.06	15.67 ± 1.01
	6		19.46 ± 1.22	18.57 ± 0.20	17.54 ± 1.48	18.29 ± 0.98
Ethanol	0	FG	1.03 ± 0.17	1.47 ± 0.78	2.09 ± 0.85	n.d.^§^
(mg/g)	2		6.76 ± 0.46 ^a^	7.18 ± 1.53 ^a^	8.27 ± 2.31 ^a^	n.d.^§^
	4		9.09 ± 1.39 ^a^	10.15 ± 0.05 ^a^	10.24 ± 0.57 ^a^	n.d.^§^
	6		10.40 ± 0.52 ^a^	10.85 ± 0.06 ^a^	11.15 ± 0.14 ^a^	n.d.^§^
	0	FGN	0.49 ± 0.01	0.44 ± 0.04	0.44 ± 0.11	n.d.^§^
	2		1.49 ± 0.01 ^b^	1.72 ± 0.00 ^b^	2.38 ± 0.41 ^b^	n.d.^§^
	4		3.77 ± 0.66 ^b^	3.48 ± 0.32 ^b^	5.53 ± 0.30 ^b^	n.d.^§^
	6		7.15 ± 1.77 ^b^	5.88 ± 0.18 ^b^	9.10 ± 0.21 ^b^	n.d.^§^

FG: Flour added with glucose, FGN: Flour added with glucose and NaCl. Values with different superscripts uppercase letter ^(A–C)^ within the same raw are significantly different (*P* < 0.01). For each parameter, values with different superscripts lowercase ^(a–b)^ letter within the same column at the same time are significantly different (*P* < 0.01). * n.d.: not detectable count, below 1 log CFU/g; ^§^ n.d.: not detectable limit, below 0.1 mg ethanol/g dough.

**Table 3:** Kinetic parameters (Leavening rate, lag leavening time, maximum CO_2_ production) of dough samples leavened with different *Z. mobilis* strains (DSM 3580, 424 and 473) in presence of glucose only (FG) or glucose and NaCl (FGN), as computed by DMFit [17] (model R^2^ also reported).

Parameter	Dough	*Z. mobilis* Strain		
		DSM 3580	DSM 424	DSM 473
Leavening Rate	FG	34.88 ± 0.18 ^A^	38.37 ± 0.96 ^AB^	46.15 ± 3.70 ^B^
(mL/h)	FGN	16.69 ± 0.46 ^A^	10.89 ± 0.335 ^B^	19.53 ± 1.28 ^C^
Lag leavening	FG	0.96 ± 0.01	0.75 ± 0.01	0.80 ± 0.12
time (h)	FGN	2.20 ± 0.28 ^A^	1.50 ± 0.54 ^B^	1.70 ± 0.17 ^AB^
Max. CO_2_	FG	97.59 ± 0.81	101.26 ± 0.89	107.38 ± 6.50
production (mL)	FGN	-	-	-
R^2^	FG	0.995 ± 0.000	0.990 ± 0.001	0.990 ± 0.003
	FGN	0.992 ± 0.002	0.994 ± 0.007	0.998 ± 0.001

Values with different superscripts uppercase letter ^(A–C)^ within the same raw are significantly different (*P* < 0.01).

## Appendix A

**Table A1:** Chimico-physical and reological properties reported in technical data sheet of the “00 Rapid” wheat flour.

Properties	Content	Method
Humidity (%)	<15.5	Buhler method
Ash (%)	<0.55	ISS1967
Protein (%)	>11	NIR
Gluten (%)	>19	ICC 106
Hagberg Index (s)	>250	ISO 3093
W 10^−4^ J	240/250	ISO 5530/4
P/L 10^−4^ J	0.45/0.65	ISO 5530/4
